# Störung der Mikrozirkulation bei COVID-19

**DOI:** 10.1007/s00063-021-00842-z

**Published:** 2021-08-10

**Authors:** Alexandros Rovas, Philipp Kümpers

**Affiliations:** Medizinische Klinik D, Allg. Innere Medizin und Notaufnahme sowie Nieren- und Hochdruckkrankheiten und Rheumatologie, Universitätsklinikum Münster, Albert-Schweitzer-Campus 1, 48149 Münster, Deutschland

**Keywords:** Sublinguale Mikroskopie, Endotheliale Glykokalyx, Endotheliopathie, SARS-CoV-2, Mikrovaskuläre Dysfunktion, Sublingual microscopy, Endothelial glycocalyx, Endotheliopathy, SARS-CoV-2, Microvascular dysfunction

## Hintergrund

Mit zunehmendem Verständnis der komplexen Pathophysiologie und der Dauer der Pandemie wird immer klarer, dass Coronavirus-Krankheit-2019 (COVID-19) mehr als nur eine virale Pneumonie ist. Konkret entwickeln COVID-19-Patienten nicht nur eine isolierte Lungenentzündung, die teilweise in einem akuten Atemnotsyndrom (ARDS) gipfelt, sondern weisen auch eine Vielzahl extrapulmonaler Symptome und Organversagen auf, wie z. B. akute Nierenschädigung (AKI), Herzschädigung, Koagulopathie und thromboembolische Komplikationen bis zum Kreislaufschock [3]. Frühe *In-vitro*-Experimente und postmortale Studien deuteten an, dass SARS-CoV‑2 (engl. „severe acute respiratory syndrome coronavirus type 2“) möglicherweise an vaskulär exprimierte ACE2(angiotensinkonvertierendes Enzym 2)-Rezeptoren bindet und so direkt die Endothelzellen befallen kann [2]. Diese Vermutung und weitere klinische Befunde nährten die Hypothese, dass es sich bei COVID-19 primär um eine (mikro)vaskuläre Erkrankung handelt, bei der das Endothel und die Mikrozirkulation eine zentrale Rolle in der Pathophysiologie und für den klinischen Verlauf spielen könnten.

Das Endothel und insbesondere seine schützende, kohlenhydratreiche Schicht – die endotheliale Glykokalyx (eGC) – regulieren die Homöostase der Mikrozirkulation [9]. In mehreren Tiermodellen ist bereits gezeigt worden, dass die Schädigung der eGC eine zentrale Rolle bei akuten und chronischen vaskulären Entzündungszuständen spielt. Beispielsweise scheint in der bakteriellen Sepsis die systemische Schädigung der eGC ein wesentlicher, vielleicht sogar der initiale Trigger für die Organdysfunktion (insbesondere akute Lungen- und Nierenschädigung) zu sein. Tatsächlich konnte in einem experimentellen Sepsismodel gezeigt werden, dass durch die Hemmung des enzymatischen Glykokalyxabbaus das Auftreten einer akuten Lungenschädigung vollständig verhindert werden kann [8].

## Material und Methoden

Um die mikrovaskuläre bzw. endotheliale Schädigung bei COVID-19-Patienten genauer zu quantifizieren, führten wir eine multizentrische prospektive Studie durch. Insgesamt 23 COVID-19-Patienten, davon 14 unter invasiver Beatmung, wurden eingeschlossen und über einen Zeitraum von 60 Tagen oder bis zur Entlassung aus dem Krankenhaus nachverfolgt. Primärer Endpunkt war die 60-Tage-Krankenhaussterblichkeit, sekundäre Endpunkte waren die Entwicklung eines ARDS oder das Auftreten thromboembolischer Ereignisse. Als Kontrollen dienten 15 gesunde Probanden. Bei allen Studienteilnehmern erfolgten die Bestimmung von a priori ausgewählten Endothel- und Glykokalyxmarkern im Plasma sowie eine Visualisierung und Analyse der sublingualen Mikrogefäße mittels Intravitalmikroskopie.

Hierbei nutzen wir eine Sidestream-Darkfield-Kamera in Kombination mit einer speziell entwickelten Software (GlycoCheck®-System; Microvascular Health Solutions Inc., Alpine, UT, USA), um die Glykokalyxdicke sublingualer Kapillaren zu quantifizieren. Spezifisch kann damit direkt am Patientenbett die „perfused boundary region“ (PBR) errechnet werden. Die PBR entspricht dabei vereinfacht gesagt der Eindringtiefe der Erythrozyten in die endotheliale Glykokalyx. Eine niedrige PBR entspricht einer intakten Glykokalyx – hohe Werte sprechen für eine zunehmend zerstörte Glykokalyx. In eigenen Vorarbeiten an Notaufnahme- und Intensivpatienten konnten wir sowohl eine sehr gute und untersucherunabhängige Reproduzierbarkeit der Methode [4] sowie ihre hohe Präzision nachweisen [1, 7].

### Neuartige Analyse der Kapillardichte per Durchmesserklassen

Neben der PBR lässt sich mit dem System auch die Dichte der sublingualen Gefäße (engl. „capillary density“) bestimmen. Dabei nutzen wir eine kürzlich erstmals publizierte Auswertungsroutine, die eine hochquantitative Analyse der Kapillardichte in einzelnen Durchmesserklassen (1 µm-Schritte im Bereich von 4–25 µm Gefäßdurchmesser) ermöglicht [6].

## Ergebnisse und Diskussion

In der Originalarbeit „Microvascular dysfunction in COVID-19 patients: the MYSTIC study“ konnten wir erstmals detailliert die Kapillarschädigung bei COVID-19 beschreiben und zeigen, dass die Patienten eine schwere Abnahme der Kapillardichte und eine ausgeprägte Schädigung der eGC aufweisen [5].

### Dramatische Abnahme der Kapillardichte bei COVID-19

**Figure 1:**
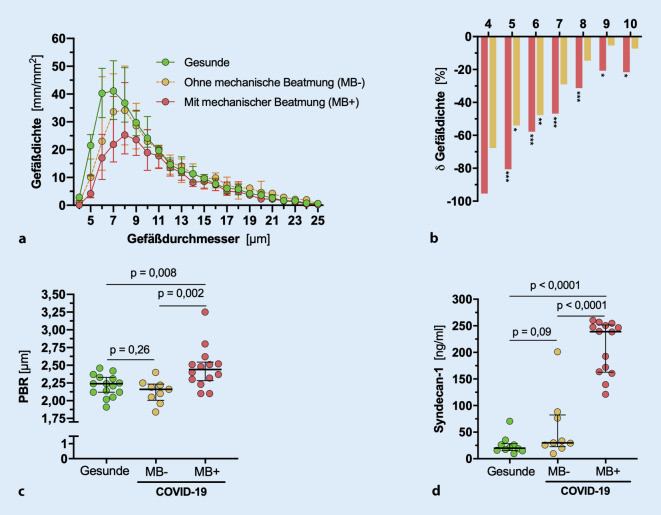

Unsere neuartige Analyse ergab eine dramatische Abnahme der Kapillardichte von bis zu 90 % im Vergleich zu gesunden Kontrollen, die sich ganz überwiegend an den kleinen Kapillaren (<10 µm) abspielte und gut mit der Krankheitsschwere korreliert (Abb. 1a).

Mechanisch beatmete Patienten wiesen dabei eine schwerere eGC-Schädigung auf, die mit erhöhten Plasmaspiegeln abgelöster Glykokalyxbestandteile (Syndecan‑1 und Hyaluronsäure) einherging (Abb. 1b–d).

### eGC-Dicke sagt die Krankenhausmortalität voraus

**Figure 2:**
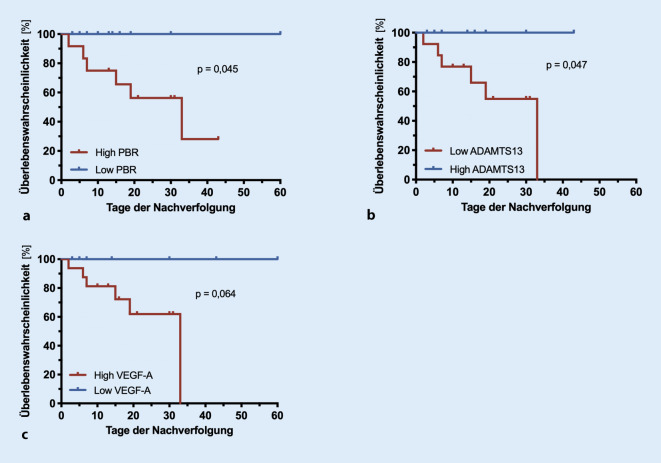

Interessanterweise konnten die Dicke der sublingualen eGC (PBR), die zirkulierenden Spiegel von ADAMTS13-Spiegel (von-Willebrand-Faktor-spaltende Protease) und von vaskulärem endothelialem Wachstumsfaktor A (VEGF-A) die 60-Tage-in-hospital-Mortalität vorhersagen (Abb. 2).

Syndecan‑1, die PBR und endotheliale Marker, wie ADAMTS13, angiotensinkonvertierendes Enzym 2 (ACE2), D‑Dimere und VEGF‑A, konnten die Entwicklung eines mittelschweren bis schweren ARDS im Krankenhausaufenthalt voraussagen. Neben den D‑Dimeren waren lediglich die PBR und die Syndecan-1-Spiegel prädiktiv für thromboembolische Ereignisse.

### Potenzielle Rolle der Angpt/Tie2-Systems bei COVID-19

Basierend auf früheren Arbeiten unserer Gruppe untersuchten wir auch die potenzielle Rolle des Angiopoietin(Angpt)/Tie2-Systems in der aktuellen Studie. Mechanistisch gesehen vermittelt die Bindung von zirkulierendem Angpt‑2 am endothelial exprimierten Tie2-Rezeptor die Aktivierung und Sekretion von Heparanase aus bestimmten zellulären Speicherpools mit konsekutivem enzymatischem Abbau der Glykokalyx [1]. Tatsächlich war Angpt‑2 (aber auch weitere Marker einer akuten Endothelschädigung) bei den COVID-19-Patienten im Vergleich zu den Kontrollen signifikant erhöht (vgl. Boxplot im Onlinezusatzmaterial).

## Resümee und Ausblick

Obwohl unsere Daten keine Kausalität beweisen können, ist es sehr wahrscheinlich, dass COVID-19 einen ausgeprägten vaskulären Phänotyp aufweist oder sogar eine neuartige vaskuläre Multisystemerkrankung darstellt. Eine genaue Quantifizierung der endothelialen Glykokalyxschäden könnte ein neuer Parameter für die Ergebnisvorhersage sein. Zukünftige Vorhersagemodelle und therapeutische Ansätze sollten daher die Bedeutung des vaskulären Endothels und seiner Glykokalyx bei COVID-19 berücksichtigen.

## Fazit für die Praxis

- Höchstwahrscheinlich handelt es sich bei COVID-19 (Coronavirus-Krankheit-2019) um eine systemische (mikro)vaskuläre Erkrankung.
- Mittelschwere und schwere Fälle von COVID-19 werden von Veränderungen der Mikrozirkulation und der endothelialen Glykokalyx begleitet.
- Künftige therapeutische Ansätze sollten die Bedeutung der Gefäßbeteiligung bei COVID-19 berücksichtigen bzw. überprüfen.

## Supplementary Information




