# Supplementary material for: Impairment of the microculation in COVID-19
Source: Med Klin Intensivmed Notfmed. 2021 Aug 10;116(6):530–4. [Article in German] doi: 10.1007/s00063-021-00842-z (PMC8353926; doi:10.1007/s00063-021-00842-z)
Supplement: Supplementary file 1 [file 63_2021_842_MOESM1_ESM.pdf]

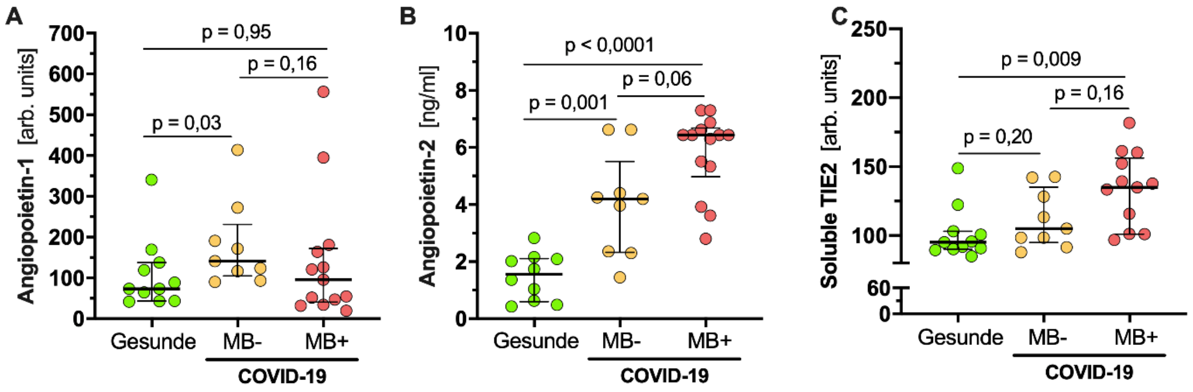

Boxplots von a) Angiopoietin-1, b) Angiopoietin-2, c) löslichem Tie2-Rezeptor zwischen gesunden Kontrollen und COVID-19-Patienten mit (rot) und ohne (orange) mechanische Beatmung (MB). Mod. nach [5]
